# Racial Differences in Paraoxonase-1 (PON1): A Factor in the Health of Southerners?

**DOI:** 10.1289/ehp.0900569

**Published:** 2009-03-12

**Authors:** Kimberly A. Davis, J. Allen Crow, Howard W. Chambers, Edward C. Meek, Janice E. Chambers

**Affiliations:** 1 Center for Environmental Health Sciences, College of Veterinary Medicine and; 2 Department of Entomology and Plant Pathology, Mississippi State University, Mississippi State, Mississippi, USA

**Keywords:** atherosclerosis, cardiovascular disease, C-reactive protein, health disparities

## Abstract

**Background:**

The southern United States (excluding Florida) has the highest age-adjusted rate of cardiovascular disease (CVD) in the country, with African Americans having a higher prevalence of CVD than Caucasians. Paraoxonase-1 (PON1), an enzyme associated with high-density lipoprotein particles, participates both in the hydrolysis of oxidized lipids (thus protecting against atherosclerosis) and in the hydrolysis of organophosphates. Higher paraoxonase activity has been associated with lower risk of atherosclerosis.

**Objectives:**

In this study we characterized the distribution of the functional *PON1*_Q192R_ polymorphisms (PON status as assessed by diazoxonase to paraoxonase ratios) and the PON1 activity levels in 200 adult males and females of both races (50 in each race/sex class) from the southern United States from commercially obtained blood bank serum samples.

**Methods:**

We used spectrophotometric methods with serum to determine PON1 status, arylesterase activities (phenyl acetate hydrolysis), and levels of cotinine and C-reactive protein (CRP).

**Results:**

African Americans had higher paraoxonase activities but lower diazoxonase activities than did Caucasians, consistent with African Americans having a lower proportion of the functional genotype QQ (QQ 15%, QR 34%, RR 44%, 7% indeterminate), than did Caucasians (QQ 60%, QR 31%, RR 7%, 2% indeterminate). Cotinine levels indicated that all samples came from non-smokers and that CRP levels were higher in African Americans than in Caucasians and higher in females than in males. CRP levels showed no association with paraoxonase activities.

**Conclusions:**

These data present initial observations for use in characterizing the poorer cardiovascular health status of the population in the southern United States and more specifically southern African Americans.

Paraoxonase-1 (PON1; EC 3.1.8.1) is an enzyme that has three important known functions: an antioxidative function in preventing the formation of oxidized lipoproteins (Shih et al. 1998), a hydrolytic function on the active metabolites of some organophosphate insecticides (e.g., paraoxon and diazoxon) and other xenobiotic substrates (van Himbergen et al. 2006), and a hydrolytic function that degrades quorum sensing factors of *Pseudomonas aeruginosa*, thereby increasing host resistance to the bacteria (Ozer et al. 2005; Stoltz et al. 2008). PON1 is synthesized primarily in the liver and is secreted into the blood, where it is associated with high-density lipoproteins (HDLs) (Costa et al. 2005; Deakin et al. 2002; James and Deakin 2004; Sorenson et al. 1999). PON1 belongs to a family of calcium-dependent lactonases/hydrolases that also include PON2 and PON3 (Gaidukov et al. 2006). The three enzymes hydrolyze aromatic and long-chain aliphatic lactones, but PON2 and PON3 lack paraoxonase (POase; phosphotriesterase) and arylesterase (phenyl acetate hydrolysis) activities (Costa et al. 2005). Lactones, such as those formed from the enzymatic and nonenzymatic oxidation of arachidonic acid and docosa-hexaenoic acid, are the endogenous substrates of PON1 (Draganov et al. 2005). PON1’s physiologic activity is thought to be that of a lipolactonase whose activity results in the prevention of atherosclerosis (Gaidukov et al. 2006). PON1 prevents the formation of oxidized low-density lipoproteins (LDLs) and protects phospholipids in HDLs from oxidation (Costa et al. 2005).

The *PON1* gene contains a variety of single-nucleotide polymorphisms in the promoter and the coding sequences (Chen et al. 2005). Previously, the *PON1*_Q192R_ polymorphism in the coding sequence was thought to influence susceptibility to cardiovascular disease (CVD) (Costa et al. 2005). The *PON1*_192_ polymorphism affects PON1’s hydro lytic activity toward several nonphysiologic substrates and thus was thought to influence its ability to protect against LDL oxidation. The Q alloform was believed to be more protective of cardiovascular health than the R alloform because it had a greater capacity to metabolize oxidized lipids (Costa et al. 2005). However, a meta-analysis of 43 published studies revealed only a very weak association of the *PON1*_192_ polymorphism with coronary heart disease (Wheeler et al. 2004) and concluded the association was of uncertain significance. Gaidukov et al. (2006) reported that the R alloform of PON1 bound HDL with a higher affinity than did the Q alloform and thus exhibited increased stability and lipolactonase activity. Bhattacharyya et al. (2008) found patients with the QQ genotype had an increased incidence of major cardiac events. Others have reported no association of PON1 genotype with CVD (Gardemann et al. 2000; Ombres et al. 1998). Thus, the association, if any, of the *PON1*_192_ polymorphism with atherosclerosis remains uncertain. Other studies have shown an association of lower POase activity with atherosclerotic diseases (Ayub et al. 1999; Jarvik et al. 2000; Mackness et al. 2001), thus demonstrating the importance of determining the phenotype (i.e., actual enzyme activity or enzyme concentration) and not just the genotype when studying atherosclerosis. Therefore the continued investigation of both genotype and phenotype in more populations at risk of CVD is warranted.

Plotting the rates of diazoxon hydrolysis [diazoxonase (DZOase)] versus paraoxon hydrolysis (POase) (i.e., DZOase/POase ratio) separates individuals into three functional genotypes of PON1 activity: *PON1*_192QQ_, *PON1*_192QR_, and *PON1*_192RR_ (Richter and Furlong 1999). However, both genotype and phenotype (i.e., overall PON1 activity) are important for determining the relationship of PON1 polymorphisms with susceptibilities to disease, sensitivity to organophosphate insecticides, and pharmacokinetic status of drug metabolism (Richter et al. 1999). Because arylesterase activity, using phenyl acetate as the substrate instead of the more toxic organophosphates as substrates, is frequently monitored as an index of PON1 activity, we also determined arylesterase activity in this study.

The significance of PON1 to cardiovascular health and sensitivity to organophosphate insecticides has been addressed experimentally through the use of *PON1* knockout mice, which were more susceptible to atherosclerosis, had HDL and LDL particles that were more susceptible to *in vitro* oxidation, and had HDL particles that were inefficient for hydrolysis of oxidized LDL particles *in vitro* (Shih et al. 1998). HDL particles isolated from transgenic mice with human PON1 had an enhanced ability to protect LDL particles from oxidation (Tward et al. 2002).

The southern United States (except Florida) has higher annual age-adjusted mortality rates from CVD (e.g., coronary heart disease and stroke) than do other regions of the country (American Heart Association 2005), and African Americans have higher annual age-adjusted mortality rates of CVD than do Caucasians. The well-characterized critical risk factors for CVD are hypertension, dyslipidemias (e.g., high LDL cholesterol and low HDL cholesterol), smoking, diabetes mellitus, a family history of disease, and obesity. Several theories have been advanced to explain the higher stroke mortality rate in the South, including low socioeconomic status, an increased prevalence of severity of hypertension, and the presence of environmental toxicants (Perry and Rocella 1998). Data on the frequency distribution of *PON1*_192_ genotypes have been reported on numerous populations, but not specifically the American southern populations, which have the worst American health statistics. Previous studies reported that African Americans have a weighted *PON1*_Q192_ allele frequency of 0.37 and a weighted *PON1*_R192_ allele frequency of 0.63, whereas Caucasians have a weighted *PON1*_Q192_ allele frequency of 0.73 and a weighted *PON1*_R192_ allele frequency of 0.27 (Chen et al. 2003; Scacchi et al. 2003). Knowledge of PON1 enzymatic activities as related to genotype, phenotype, race, sex, and age within a population may provide a useful explanation for some of the disparities in CVD among demographic groups.

To characterize the functional genotype distribution (as assessed by DZOase/POase ratios) and activity levels of PON1 as contributors to the higher CVD in southern populations, in the present study we investigated PON1 activity levels with three substrates (paraoxon, diazoxon, and phenyl acetate) in the serum of African-American and Caucasian southerners within race, sex, and age groups. In addition, we also investigated the levels of the nicotine metabolite cotinine (as a possible influence of cigarette smoking on PON1 activity and concentrations) (James et al. 2000; Nishio and Watanabe 1997) and the inflammatory marker C-reactive protein (CRP), a biomarker for increased risk of coronary heart disease (reviewed by Casas et al. 2008). Our study samples came from a commercial vendor that had obtained serum samples from blood banks in Alabama and Tennessee. We obtained a total of 200 serum samples from adult men and women in equally distributed race and sex classes who self-identified as being Caucasian or African American. With the data obtained, we observed that African Americans in the South have lower DZOase activities, higher POase activities, and lower DZOase/POase ratios than Caucasians, reflecting the significantly different distribution of QQ, QR, and RR functional genotypes observed in African Americans than that found in Caucasians. Our laboratory is expanding these studies at present with samples from individuals whose cardiovascular health status is known to determine what role the difference in frequency distribution of QQ, QR, and RR functional genotypes and differences in activity levels between the racial groups may play in the CVD health disparities observed and to investigate the associations that might exist with exposure to environmental chemicals.

## Materials and Methods

### Chemicals and samples

We purchased all biochemicals from Sigma Chemical Company (St. Louis, MO). We synthesized paraoxon as described previously by Chambers et al. (1990). Diazoxon was purchased from ChemService (West Chester, PA). Serum cotinine was determined using the Cotinine Direct ELISA (Calbiotech, Inc., Spring Valley, CA). Serum CRP was determined using a high-sensitivity CRP ELISA (Calbiotech, Inc.). We purchased serum samples from Integrated Laboratory Services-Biotech (Chestertown, MD), which had obtained serum samples from blood banks in Alabama and Tennessee. We excluded from the study individuals who were < 25 years of age or > 65 years of age, who were known or suspected to be infected with HIV (human immunodeficiency virus) or hepatitis, or who did not self-declare as Caucasian or African American; no demographic information was available on any of the samples other than age, sex, and self-declared race. The mean ages (± SD) were 33.5 ± 6.9 years for African-American females, 40.4 ± 8.5 years for African-American males, 39.3 ± 9.2 years for Caucasian females, and 36.4 ± 8.5 years for Caucasian males. The Institutional Review Board for the Protection of Human Subjects in Research at Mississippi State University approved the study protocol.

### POase assay

We measured paraoxon hydrolysis spectrophotometrically in micro-titer plates according to a method described by Richter and Furlong (1999). We incubated paraoxon (1.2 mM final concentration; stock solution in dry ethanol) in paired serum samples of 1 μL serum in a reaction volume of 200 μL. A calcium buffer solution of Tris-HCl (0.1 M, pH 8.0), 2 mM CaCl_2_, and 2 M NaCl was used to activate PON1 in one set of triplicate samples made from the same three independent dilutions of serum. We used an EDTA buffer solution with 1 mM EDTA instead of CaCl_2_ to eliminate PON1 activity in the paired set of triplicate samples made from three independent dilutions of serum. After mixing and incubation at 37°C for 5 min, paraoxon was added, mixed, and incubated at 37°C for 20 min with shaking. After incubation, the enzyme reactions were terminated by adding a solution of 50 μL of 20 mM EDTA plus 2% Tris base solution in deionized water. The 4-nitrophenol released was quantified at 405 nm. We subtracted the mean of the EDTA triplicates from the mean of the CaCl_2_ triplicates for each individual to correct for non-PON1-mediated hydrolysis. Data were expressed as micromoles of paraoxon hydrolyzed per minute per liter of serum. The reaction rate was linear during the 20-min incubation time.

### DZOase assay

We measured the 2-isopropyl- 4-methyl-6-hydroxypyrimidine (IMHP) released from diazoxon hydrolysis spectrophotometrically in a continuous assay according to the method described by Richter and Furlong (1999). We incubated diazoxon (2.0 mM final concentration; solution in dry ethanol) in each sample. Diluted serum (20 μL serum plus 1,950 μL of the calcium buffer described above) was pre incubated at 37°C for 5 min, and then 20 μL diazoxon (2.0 mM final concentration) was added. The solution was mixed and immediately placed into a cuvette. We quantified the IMHP continuously for 2 min at 270 nm. Samples were run in quadruplicate and the values averaged as micromoles of diazoxon hydrolyzed per minute per liter of serum.

### Arylesterase assay

We assessed arylesterase activity using phenyl acetate as the substrate. We diluted serum to 5 μL/mL in 0.05 M Tris-HCl plus 2 M NaCl buffer (pH 8.0) containing either 2 mM CaCl_2_ to activate PON1 or 1 mM EDTA to serve as a blank. These dilutions (2.5 mL) were warmed to 37°C, and then 25 μL of 50 mM phenyl acetate in ethanol was added. Absorbance was measured at 270 nm for 3 min, and the slopes were calculated. PON1 activity was expressed as micromoles of phenyl acetate hydrolyzed per minute per liter of serum.

### C-reactive protein

Serum CRP was quantified spectrophotometrically at 450 nm using a high sensitivity CRP ELISA kit (solid-phase direct sandwich method) following the manufacturer’s directions and interpolating the values from a standard curve. Data were expressed as milligrams of CRP per liter of serum. We did not include CRP values > 10 mg/L in these analyses because these values are not used in assessing cardiovascular risks (American Heart Association 2005).

### Cotinine assay

We quantified serum cotinine spectrophotometrically at 450 nm using a cotinine ELISA kit following manufacturer’s directions. Data were expressed as nanograms per milliliter of serum.

### Statistical analysis

All data were analyzed using the SAS System for Windows (version 9.1; SAS Institute Inc., Cary, NC). We performed efficacy analyses using a linear mixed model (PROC MIXED), and we used the least square means when we found statistical significance to determine the differences between groups (race, age groups, and sex) with respect to the mean DZOase and POase activities. In the mixed models, the fixed effects were race, age group, and sex. All statistical comparisons were two-sided using a 0.05 significance level.

## Results

### DZOase and POase activities, and DZOase/POase ratios

**Figure 1:**
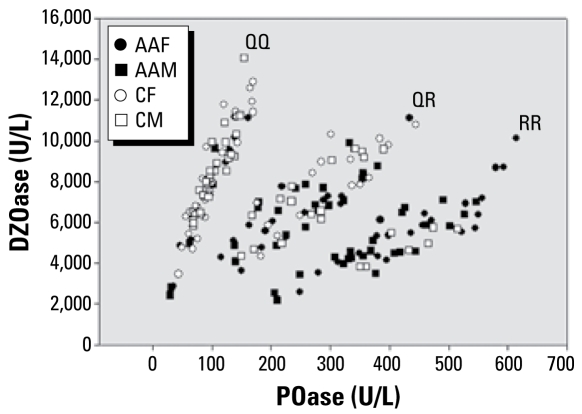
DZOase activities versus POase activities in sera of male and female Caucasian and African-American southerners, as well as the distribution of individuals into three *PON1*_192_ functional genotypes (QQ, QR, and RR). Abbreviations: AAF, African-American female; AAM, African-American male; CF, Caucasian female; CM, Caucasian male. The sample population (*n* = 191) consisted of 47 AAFs, 46 AAMs, 49 CFs, and 49 CMs. Results from 3 AAFs, 4 AAMs, 1 CF, and 1 CF were indeterminate and thus were not included in the plot.

The overall DZOase and POase activities for all subjects and for both races are shown in Table 1, and the DZOase versus POase plots are shown in Figure 1. Similar to results of Richter and Furlong (1999), plotting hydrolytic rates separated individuals into one of three functional *PON1*_192_ genotypes for determining PON1 status. The frequency distribution of the functional genotypes for the entire population was QQ (0.375), QR (0.325), and RR (0.255). Nine data points (frequency of 0.045) were indeterminate for apparent QR and RR genotypes, seven of which were from African Americans (three females, four males) and two from Caucasians (one female, one male); these were not included in Figure 1. These nine indeterminate individuals all fell in the region between the QR and RR functional genotype. These individuals may have nonsense or missense mutations as previously described by Jarvik et al. (2002).

### Differences in race

**Figure 2:**
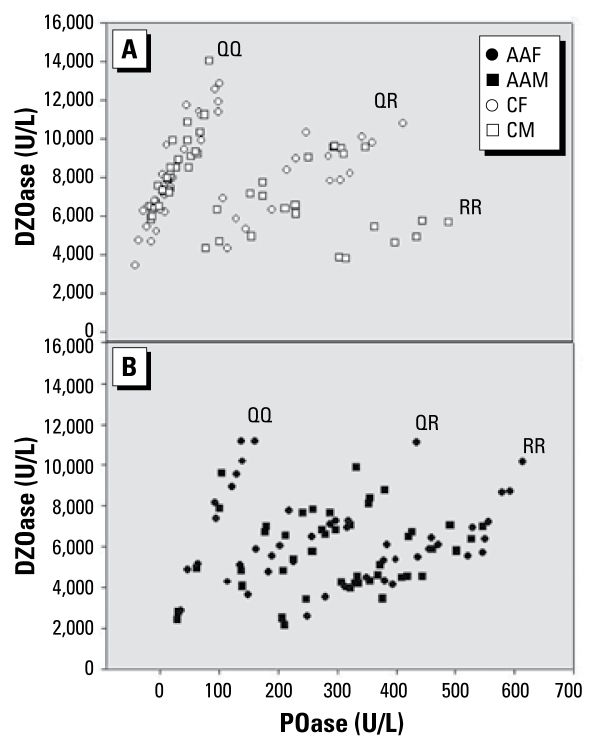
DZOase activities versus POase activities in sera of male and female Caucasian southerners (*A*) and African-American southerners (*B*). Abbreviations: AAF, African-American female; AAM, African-American male; CF, Caucasian female; CM, Caucasian male.

The frequency distribution of QQ (0.15), QR (0.34), and RR (0.44) functional genotypes within the African-American population was substantially different from the distribution in the Caucasian population [QQ (0.60), QR (0.31), and RR (0.07); Figure 2]. Caucasians had a 4-fold higher proportion (± 95% confidence interval) for the QQ genotype (0.60 ± 0.15) than did African Americans (0.15 ± 0.11), and this difference was significant. In our study population, Caucasians had a *PON1*_192Q_ allele frequency of 0.77 and a *PON1*_192R_ allele frequency of 0.23. African Americans had a *PON1*_192Q_ allele frequency of 0.34 and a *PON1*_192R_ allele frequency of 0.66.

The mean DZOase activities of African Americans were significantly lower than those of Caucasians (*p* = 3.25^−08^), whereas the mean POase activities of African Americans were significantly higher than those of Caucasians (*p* = 6.52^−09^; Table 1). The mean DZOase/POase ratio of African Americans was significantly lower (*p* < 0.05) than the ratio of Caucasians (Table 1). The racial differences in the DZOase activity, the POase activity, and the DZOase/POase ratio are all to be expected given the differences in the functional genotype distributions and the differences in the *PON1*_192_ allele frequencies observed between Caucasians and African Americans in our study population. When we compared African Americans and Caucasians within the same functional genotype, we found no statistically significant differences in mean POase and DZOase activities. However, within the QQ genotype, we found a nonsignificant trend, with Caucasians having a higher mean DZOase activity than African Americans.

### Differences in sex and PON1_192_

The mean POase activities of all males and all females and the mean DZOase activities of all males and all females were not significantly different (Table 2). The mean DZOase/POase ratio of all males and all females was also not significantly different. In addition, we observed no differences between sexes in activities and the DZOase/POase ratios within each race. However, the mean POase activities, the mean DZOase activities, and the mean DZOase/POase ratios were significantly different in African-American females compared with Caucasian females and in African-American males compared with Caucasian males (Table 2), again likely reflecting the difference in functional genotype distribution between the racial groups.

### Differences in age and PON1_192_

**Figure 3:**
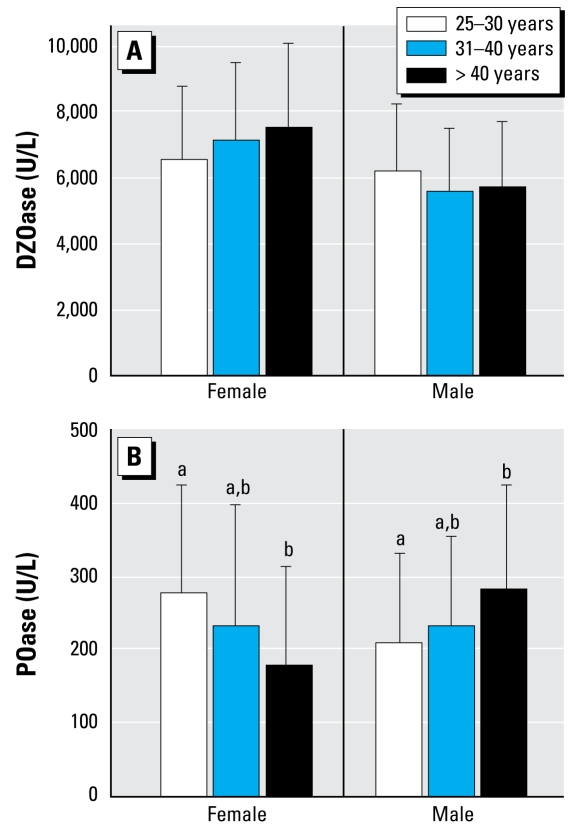
DZOase (*A*) and POase (*B*) activities in three age classes of male and female southerners. Female age groups: 25–30 years (*n* = 32), 31–40 years (*n* = 36), > 40 years (*n* = 32); male age groups: 25–30 years (*n* = 28), 31–40 years (*n* = 27), > 40 years (*n* = 45). In (*B*) within a sex, means shown with different lower case letters are significantly different (*p* < 0.05).

We separated individuals into three age groups (25–30 years, 31–40 years, and > 40 years) for statistical analysis. In females, POase activities appeared to be higher (*p* = 0.0366) in the youngest group (*n* = 32) compared with the oldest group (*n* = 32) (Figure 3). The reverse appeared to be true in males, with POase activities lower (*p* = 0.0209) in the youngest group (*n* = 28) compared with the oldest group (*n* = 45). However, these differences are probably the result of uneven sampling of functional genotypes among these arbitrary age ranges. The females had QQ distributions of 27%, 47%, and 63% in the youngest, middle, and oldest age groups, respectively. Conversely, the males had QQ distributions of 46%, 32%, and 25% in the youngest, middle, and oldest age groups, respectively. We found no significant differences in DZOase activities among the three age groups.

### Arylesterase activity

**Figure 4:**
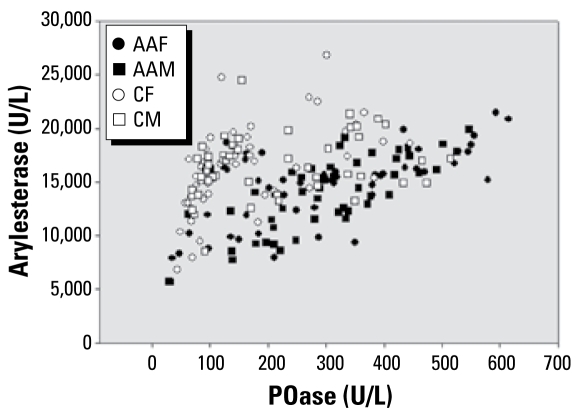
Arylesterase activity as monitored with phenyl acetate versus POase activities in sera of male and female African-American and Caucasian southerners. Abbreviations: AAF, African-American female; AAM, African-American male; CF, Caucasian female; CM, Caucasian male.

Results plotting phenyl acetate hydrolysis (arylesterase activity) versus POase activity showed a trend toward separating individuals into the three functional genotypes (Figure 4), but did not yield as clear-cut a distinction among the three functional genotypes as did the plot of POase versus DZOase. Caucasians had slightly higher activities (16,407 ± 341 U/L) than African Americans (14,276 ± 327 U/L), but this was not statistically significant.

### Cotinine levels

Serum cotinine levels were less than the levels indicative of active smokers (> 78 ng/mL; Wall et al. 1988) for all samples.

### CRP levels

CRP levels were significantly higher in African Americans (4.70 ± 3.50 mg/L) than in Caucasians (3.71 ± 2.60 mg/L; *p* < 0.05) and higher in females (4.93 ± 3.20 mg/L) than in males (3.53 ± 2.80 mg/L; *p* < 0.05), similar to previously published data (Lakoski et al. 2006). We found no significant association between POase and CRP levels or DZOase and CRP levels. Individuals with CRP levels > 10 mg/L were not included in this analysis because CRP levels > 10 mg/L are not considered reliable for indicating increased risk of CVD.

## Discussion

PON1 plays an important role in hydrolyzing many substrates, including oxidized lipids and active metabolites of organophosphate insecticides (Chambers 2008). The single-nucleotide polymorphism at position 192 has been proposed to have a significant effect on PON1 hydrolytic activity, xenobiotic metabolism, and the onset of CVD, with the R alloform earlier considered to be associated with vascular disease (Harel et al. 2004). Despite the fact that southerners (except Floridians) in general and African Americans in particular have higher annual age-adjusted mortality rates from CVD than do people in other regions of the United States, data on the distribution of *PON1*_Q192R_ in the serum of Caucasians and African Americans in the South have not been documented.

In the present study we determined the *PON1*_192_ functional genotype distribution (as assessed by DZOase/POase ratios) and PON1 enzymatic activities (paraoxon and diazoxon hydrolysis, phenyl acetate hydrolysis) in the serum of 200 African-American and Caucasian southerners. We assumed that the individuals sampled from Alabama and Tennessee blood banks were residents of the South and therefore representative of southerners in general. This study revealed significant differences in the functional *PON1*_192_ genotype distribution between Caucasians and African Americans. Caucasians had an overwhelmingly higher distribution of the functional QQ genotype (60%) than did African Americans (15%). Our study agrees with results of Chen et al. (2003) and Scacchi et al. (2003), who reported that African Americans have a higher R allele frequency (0.63) and Caucasians have a higher Q allele frequency (0.73). However these two studies did not report the activity level (phenotype), which is ultimately more important in functional capacity to hydrolyze oxidized lipids.

The arylesterase activity plot, where phenyl acetate was substituted for diazoxon, did not yield as straightforward a separation of functional genotypes as did the paraoxon versus diazoxon plot. Most laboratories report on aryl esterase activity using phenyl acetate, probably because of the high cost, toxicity, and instability of diazoxon. However, phenyl acetate is not as useful as the toxic organophosphate substrate for characterization of PON1 status. Although the African Americans had a significantly lower mean DZOase activity than did Caucasians, the lower activity of African Americans with phenyl acetate was not statistically significant. Probably because of the low numbers after the population was divided into functional genotypes, we found a trend within the QQ functional genotype (not statistically significant) that Caucasians had higher DZOase activity; this suggests that phenotype is important in characterizing risk factors and indicates that more research with larger groups of known cardiovascular health status would be useful.

Much of the difference in DZOase and POase activities between the races probably resulted from the difference in the *PON1*_192_ genotype distribution; however, racial differences in other genotypic influences (e.g., promoter polymorphisms that influence expression) and in environmental and lifestyle influences, (e.g., diet, statins and other drugs, exposure to environmental factors) also probably contribute to the difference. However, the cotinine levels indicated that none of the serum samples came from smokers, so smoking was not an influence on the PON1 levels measured.

Differences in PON1 activities between the sexes have not been shown to be significant in other studies (Mueller et al. 1983). Results from our study also do not indicate a significant difference when comparing DZOase and POase activities of females and males separately or within each race.

In the present study we found that with age, POase activity appeared to decrease in females but increase in males, whereas DZOase activity did not change significantly with age in either sex. The difference in POase activity just reaches significance but is likely the result of uneven sampling of functional genotypes within the various age ranges, as mentioned in the “Results.” This interpretation emphasizes the need to consider potential confounders (in this case, functional genotype sampling disparities among age classes) when drawing conclusions about differences in activity levels among groups of individuals. Other studies have shown that serum PON1 activity is lower during development and at birth but increases over time (Chen et al. 2003). PON1 activities have also been reported to remain constant during adulthood or decrease in elderly subjects (Costa et al. 2005; Jarvik et al. 2002; Milochevitch and Khalil 2001).

The individuals sampled here did not appear to be smokers, as indicated by the low cotinine levels. Although we do not know whether the blood banks screened out smokers, it would be highly unlikely to obtain 200 random samples with no smokers among them by chance. Because smoking can affect PON1 levels, we can assume that this potential factor did not influence the PON1 activity levels measured here. Certainly other xenobiotics may have influenced the activity levels, but we had no knowledge of drugs (e.g., statins) or environmental chemicals to which these individuals may have been exposed.

Although we did not know the health status of the individuals in this study, we did determine CRP concentrations as a biomarker of inflammation and CVD. We did not include individuals with CRP levels > 10 mg/L in these calculations because recommendations of the American Heart Association and the Centers for Disease Control and Prevention suggest excluding these CRP concentrations from clinical decisions (Pearson et al. 2003). We found that females had higher CRP concentrations than did males (*p* < 0.05) and that African Americans had higher CRP concentrations than did Caucasians (*p* < 0.05). These results on sex and race are consistent with literature reports (Kelley-Hedgepeth et al. 2008; Lakoski et al. 2006). CRP is influenced by modifiable risk factors such as body mass index, exogenous estrogen, diabetes, hypertension, smoking, alcohol use, HMG-CoA (3-hydroxy-3-methylglutaryl-coenzyme A) reductase inhibitors, aspirin use, physical activity, LDL, and HDL (Cushman et al. 1999; Ridker et al. 1999). However, we have no information regarding these factors in our study population.

One potential confounder worth noting is the potential inaccuracy of the self-identification of race. Although individuals may be primarily one race, in many cases there has been racial mixing in the past that may or may not be known to the individuals, so the racial designation may not have been totally accurate.

## Conclusions

In this article we report the *PON1*_192_ functional genotype distribution, PON1 enzymatic activities (POase, DZOase, and arylesterase), and mean DZOase/POase ratios in the serum of African-American and Caucasian southerners, along with the CRP and cotinine levels of these individuals. Most important, we found significant racial differences in the *PON1*_192_ functional genotype distribution, with African Americans—the race with higher CVD—displaying a greater proportion of the functional RR genotype. Although, at present, the relationship of the functional genotypes to CVD is equivocal, our data support the hypothesis that the functional RR genotype is less protective of cardiovascular health. These data suggest that further study using samples of southerners from both races—where cardio vascular health status, lifestyle, and environmental influences are known—would be of value in characterizing risk of both CVD and susceptibility to organophosphate insecticide toxicity. In addition, determining the complete *PON1* genotype (i.e., determining the promoter and protein polymorphisms) and quantitation of PON1 expression would allow the evaluation of what factors other than genotype contribute to the differences in PON1 activity observed between Caucasians and African Americans. Our laboratory is currently engaged in such a study with southerners of both races and both sexes where the presence of CVD and any therapeutic interventions are known.

## Figures and Tables

**Table 1:** POase and DZOase activities and DZOase/POase ratios in the serum of African-American and Caucasian southerners (mean ± SD).

Race	No.	POase activity	DZOase activity	DZOase/POase ratio
All	200	240 ± 144	6,847 ± 2,314	—
African American	100	297 ± 144*	5,973 ± 2,065*	29 ± 24*
Caucasian	100	183 ± 121	7,720 ± 2,227	60 ± 30

Data are expressed as micromoles substrate hydrolyzed per liter of serum.

* *p* < 0.05 compared with Caucasians.

**Table 2:** POase and DZOase activities and DZOase/POase ratios in the serum of African-American and Caucasian southerners by sex (mean ± SD).

Race/sex	POase activity	DZOase activity	DZOase/POase ratio
All			
Female	230 ± 154	7,069 ± 2,386	40 ± 32
Male	250 ± 134	6,023 ± 2,229	41 ± 31
African American			
Female*	300 ± 162	6,289 ± 2,193	31 ± 26
Male*	295 ± 125	5,657 ± 1,897	26 ± 21
Caucasian			
Female	161 ± 109	7,849 ± 2,335	65 ± 28
Male	206 ± 129	7,591 ± 2,129	54 ± 32

Data are expressed as micromoles substrate hydrolyzed per liter of serum of 50 individuals of each race and sex.

* *p* < 0.05 for all three values, comparing African-American females with Caucasian females and comparing African-American males with Caucasian males.
